# Anything but Shadowing! Early Clinical Reasoning in Emergency Department Improves Clinical Skills

**DOI:** 10.5811/westjem.2017.10.36691

**Published:** 2017-12-22

**Authors:** Regina Royan, Christine Wu, Nik Theyyunni, Sacha Montas, James A. Cranford, Joseph B. House, Michael P. Lukela, Sally A. Santen

**Affiliations:** * University of Michigan Medical School, Ann Arbor, Michigan; † Michigan Medicine, Department of Emergency Medicine, Ann Arbor, Michigan; ‡ Michigan Medicine, Department of Internal Medicine and Pediatrics, Ann Arbor, Michigan; § Virginia Commonwealth University School of Medicine, Richmond, Virginia

## Abstract

**Introduction:**

Transitioning from the pre-clinical environment to clerkships poses a challenge to students and educators alike. Students along with faculty developed the Clinical Reasoning Elective (CRE) to provide pre-clinical students exposure to patients in the emergency department and the opportunity to build illness scripts and practice clinical skills with longitudinal mentorship in a low-stakes environment before entering clerkships. It is a voluntary program. Each year, the CRE has received overwhelming positive feedback from students. The objective of this study is to determine if the CRE improved students’ clinical skills and reported comfort in their skills.

**Methods:**

We examined the relationships between students’ self-reported participation in the CRE and their individual scores on a comprehensive clinical assessment (CCA) at the end of the pre-clerkship period. A total of 178 students took the CCA exam in 2016. Of these, 113 participated in the CRE and 65 did not. Seven students who participated in CRE did not complete the exit survey and were omitted from analysis. We performed regression analysis and dichotomous (participants/nonparticipants) comparisons of means with t-tests. Survey of student reactions was collected.

**Results:**

Participants completed an average of 10 sessions over the course of the program (range=1–20). Involvement in the CRE was associated with significantly increased scores on Abdominal History; Pulmonary Physical Exam; Overall History-Taking; Overall Communication; and Overall Physical Exam (p<0.05). Nearly all students (97%) reported that the program offered opportunities to enhance clinical skills, increased their comfort with patients, and better prepared them for their clinical years.

**Conclusion:**

There were measurable improvements in clinical skills performance for students who participated in CRE. As many schools seek to incorporate early clinical exposure to their curricula, this program provides a successful framework to provide meaningful clinical exposure to real patients that also shows objective benefits to students’ clinical skills.

## INTRODUCTION

One of the great challenges in medical education is the ability to transition the pre-clinical learner into a clinician who is able to readily recognize, diagnose, and treat patients. Studies suggest that students can find this period stressful.^1^,^2^ In particular, students find it challenging to apply their knowledge in a practice setting and report anxiety about being evaluated on unfamiliar skills such as history-taking, performing physical examinations, proficiency in oral case presentations, and generation of differential diagnoses.^3^–^5^

The Clinical Reasoning Elective (CRE) is a student-led program designed to provide pre-clinical students exposure to patients and the opportunity to practice building differential diagnoses. The program has been a supplement to the curriculum of a U.S. medical school for the last five years, where students traditionally complete 18 months of didactic learning followed by two years of clinical experience. In this period students have a longitudinal clinical skills course that primarily uses standardized patients.

The program was designed to provide stepwise exposure to the clinical environment distinct from shadowing, in order to foster student development of self-directed learning.^6^,^7^ With this goal in mind, the elective serves as a unique opportunity for students to practice formalizing the integration of data gathering from the history and physical, synthesizing these findings into assessments, and generating plans. Students are able to practice and develop these essential clinical skills without the performance pressure or formal grading that occurs during traditional clinical clerkships. These aspects have made the program incredibly popular among students, as it is one of the first opportunities for pre-clinical students to have meaningful experiences interacting with patients on the wards and get their first taste of clinical medicine.

In the 2015–2016 academic year, 113 (of 178) second-year pre-clerkship students were matched in pairs with 55 physician-mentors in the emergency department (ED) or an inpatient setting. Students completed histories and physical examinations on patients who presented with clinical problems related to the organ systems they were currently studying in the pre-clerkship curriculum, with an emphasis on independent learning and developing illness scripts.^8^–^11^

Students were expected to evaluate patients as a pair (without faculty present), formulate a differential diagnosis, and present the findings to their faculty member. Faculty provided feedback on their history, exam, presentation, and differential diagnosis. Students were encouraged to complete a minimum of two four-hour sessions with their faculty each month; however, there was no penalty for students who completed fewer sessions. Shifts were completed during normally scheduled faculty shifts, with preference for shifts where there were not other learners paired with the faculty member. Faculty were not provided additional compensation; however, evaluations were provided to each faculty member to be used for promotion. Student pairs were partnered with the same faculty member for the duration of the program to foster a longitudinal relationship with increased clinical independence as the year progressed.

Since its inception, the CRE has received overwhelmingly positive feedback in post-experience surveys from students; however, little is known about the objective benefit of the program with respect to students’ clinical proficiency. The objective of this study was to determine if CRE improved students’ clinical skills and reported comfort in their skills compared to those who do not participate.

## METHODS

### Participants

A total of 178 students completed the Comprehensive Clinical Assessment (M2 CCA) exam in 2016. Of these, 113 participated in the CRE and 65 did not. Student participation over the course of the eight-month program was self-reported through an exit survey (106 out of 113 students responded to the exit survey; 94% overall response rate and 82–94% item-level response rate). Seven students who participated in CRE did not complete the exit surveys and were omitted from analysis since we could not determine their participation in CRE. Thus, the total number of students was 171. Students completed shifts at the following locations: Adult ED (59%); Pediatric ED (22%); Emergency Critical Care/Short Stay Unit (17%); VA ED (10%); Other Inpatient Setting (6%), usually but not always at the same location.

### Subjective Outcomes

Students completed an exit survey to provide feedback on the program including the following: 1) how many shifts they completed; 2) description of activities completed during their shifts; 3) contribution of these activities to their learning; and 4) interest in emergency medicine as a future specialty (Appendix A). The survey was designed by students (content validity evidence) and reviewed by the students as well as faculty supervisor of the program (content and response process validity evidence).^12^

### Objective Outcomes

To objectively measure the influence of participation in the CRE on students’ clinical skills, self-reported participation in the CRE was compared with students’ individual scores on the M2 CCA. We obtained institutional review board approval for this study.

The M2 CCA takes place at the end of the pre-clinical curriculum and uses standardized patient cases to assess student performance on 12 domains including the following:

1. Abdominal, Cardiac, Pulmonary, Neurology, and Musculoskeletal Physical Exam Skills
2. Abdominal and Cardiac History-taking
3. Overall Measures for Physical Exam, History-taking, Verbal Presentation, Note-writing, and Communication

The M2 CCA exam is formatted similar to the USMLE Step 2 Clinical Skills Exam, with each patient station requiring 2–3 tasks for the student to complete in an allotted time frame. The student receives the patient’s age, vital signs, and chief complaint before entering the exam room. Station tasks may include taking a history, performing a focused physical exam, writing a patient encounter note, or giving a verbal presentation to a faculty member.^13^ Students are scored with standardized rubrics by faculty and standardized patients.

### Validity Evidence

Content validity evidence for the CCA2 is based on the faculty development committee providing expert judgment for content. Response process validity evidence is provided by a plan-do-check-ask process for improvement of the cases each year^14^ reliability and equivalence of two parallel examinations that have been developed under highly defined quality assurance (QA The CCA2 results are examined and the cases adjusted to improve response process validity. In addition, the standardized patient (SP) communication scoring has significant training and inter-rater reliability testing using a kappa statistic. To quality control the SP scoring the program routinely runs kappa between raters and retrains raters scoring differently. The majority of the kappa values were > 0.6. Relationship to other variables is provided with this study (Appendix B). Finally, scores below the passing standard are independently re-scored for confirmation. If the failure is confirmed, the student must undergo remediation and re-take any failed components of the CCA. Successful completion of the M2 CCA is required for advancement to the clinical clerkships.

### Analysis

We studied the relationship between students’ scores on individual components of the CCA and the number of times they participated in the CRE. CRE participation was statistically significantly correlated with scores on five of the domains, and for these domains we conducted analyses with CRE participation treated as a continuous variable and as a binary variable. First, the influence of participation in the CRE with students’ scores was examined with participation as a continuous measure (dose-dependent variable) based on the number of sessions each student attended using bivariate regression analysis. We then dichotomized the CRE participation variable (participant in CRE vs. non-participant) and used independent samples t-tests to examine group differences on CCA scores. The demographics of students participating and not were compared. In addition, the regression analysis included gender, age and race as covariates to test the effects of demographics on the results. An alpha level of 0.05 was used for all analyses. Student responses to the exit survey are reported as descriptive statistics. The IRB approved the study.

## RESULTS

In our sample, the mean age was 27 years (*SD* = 2.6), and 54% were female. The breakdown by race/ethnicity was 6% African American, 24% Asian, 6% Hispanic, and 64% White. We examined associations between the demographic variables and participation in the CRE. Results showed that those who participated in the CRE were not statistically different in age, race/ethnicity and gender.

Students who participated in the CRE completed an average of 10 shifts with their mentors (12% of students completed 1–5 shifts; 50% of students completed 6–10 shifts; 22% completed 11–15 shifts; and 16% completed 16–20 shifts).

We conducted five separate bivariate regression analyses with the M2 CCA domain scores regressed on the number of CRE shifts a student completed. The unstandardized regression coefficients for five domains were statistically significant and provide insight into the range of impact that CRE can have on a student’s scores (Table). The table notes the effect as well on the student’s score for each CRE shift. For example, for the Abdominal History component of the exam each completed CRE shift was associated with an increase of about a third of a point in a student’s scores. The mean scores between participants and non-participants on four of the domains of the M2 CCA were statistically significant (Table).

**Figure 1:**
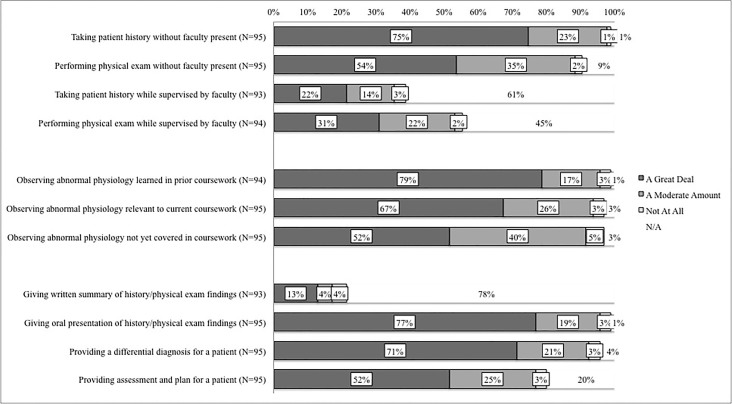
Student exit survey responses: “Of the activities that you completed during your shifts, how much did each of the following contribute to your learning?”

**Figure 2:**
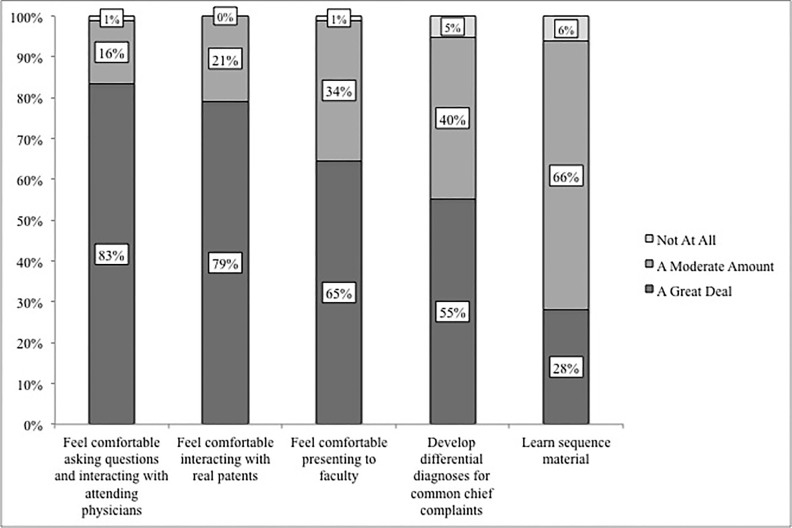
Student exit survey responses: “To what extent did the CRE help you...” (N=96).

Student activities during a typical CRE shift were elicited as part of an exit survey. Follow-up questions collected students’ opinions on how much each of those activities contributed to their learning (Figure 1). A majority of students completed patient histories and performed physical exams without faculty present and found these experiences to be highly beneficial. Students appreciated the opportunity to have real-world application and experience with conditions covered in the didactic pre-clinical curriculum. Self-reported perceived educational value of the CRE is shown in Figure 2.

One hundred percent of respondents reported that the elective increased their comfort with interacting with patients, asking questions, and interacting with attending physicians. Almost all participants (95%) reported that the CRE helped them build differential diagnoses for common chief complaints, with over half believing that this experience contributed “a great deal.” In addition, 72% of student participants rated their learning experience with their mentor as “excellent,” while 22% rated it as “Good.”

Representative narrative students’ comments demonstrate the overall positive student experience:

Student #1: *“CRE was the highlight of my preclinical education because it both reminded me of what was to come down the pipeline (i.e. practicing clinical medicine) and provided me with a much more interactive, engaging environment to learn clinical reasoning, history taking, and communication skills.”*
Student #2: “*This was my first “independent” experience being in the hospital and seeing patients. We were pushed past our comfort zone and expected to present on all our patients. Even though we were beginners we were treated like colleagues and with the utmost respect. It was a safe yet challenging environment to learn medicine and prepare for our M3 careers.”*
Student #3: “*The experience [my attending] provided in clinic serves as the framework for almost everything I learn in medical school. As the first attending to help me develop the thought process required for patient management, this contribution to my learning was invaluable.”*
Student #4: “*While this may sound absurd, I was nervous to touch patients during the first few days, especially if the patients were in pain. I now appreciate how important it is to do a complete physical examination, even if the patient is uncomfortable, and have the confidence to actually perform the exam.”*

**Figure 3:**
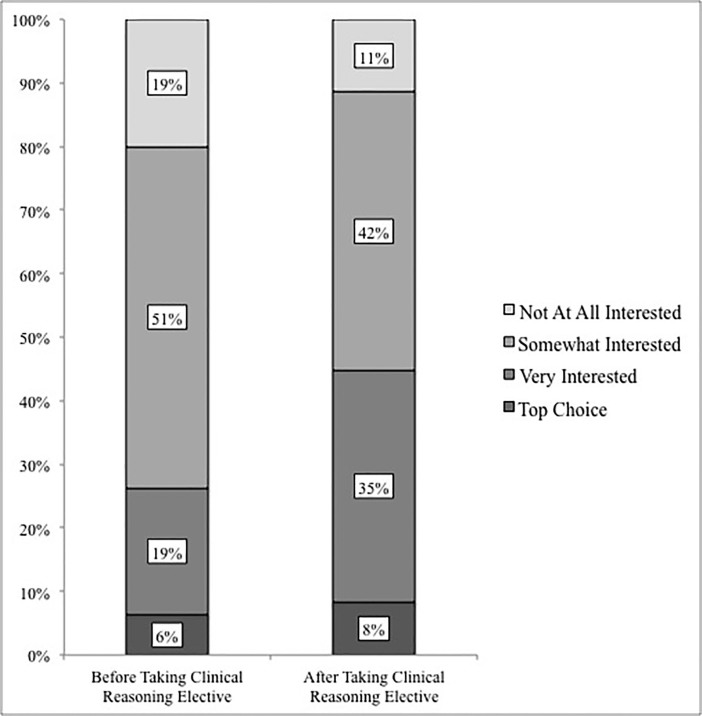
Student interest in emergency medicine before and after participation in the clinical reasoning elective.

A secondary outcome from participation in the CRE was increased student interest in EM as a specialty (Figure 3). Before taking the CRE, 26% of students were “very interested” in EM or considered it to be their “top choice.” However, after the completion of the elective, 46% of students reported that they were “very interested” in EM or considered it to be their “top choice.”

Participants were asked about their interest in EM before and after the CRE, and for both questions the response scale was 1 = My top choice, 2 = Very interested, 3 = Somewhat interested, and 4 = Not at all interested, i.e., lower scores indicated greater interest. Results from a within-subjects t-test showed that interest in EM showed a statistically significant increase from before (M = 2.9) to after (M = 2.6) the CRE, t (94) = 4.5, p < .001, with a small-to-medium effect size, Cohen’s d = 0.46.

## DISCUSSION

Participation in a CRE for medical students significantly improved student scores on the end of pre-clerkship clinical skills examination in several domains. Furthermore, students’ survey responses demonstrated that they felt more comfortable with their overall clinical skills and knowledge.

Notably, the value attributed to CRE activities by students suggests that participants gained learning experiences and early clinical exposure that are unattainable elsewhere in the standard pre-clerkship curriculum. Figure 1 highlights the value that novice learners place on the non-shadowing structure of the program that allows them to see patients on their own.

Within the ED, students are exposed to a variety of abnormal physical findings and prototypical chief complaints, which may aid in retention of clinical decision-making algorithms.^15^–^19^ Pairing the chief complaints of patients with students’ current systems of study may show even greater benefit in helping students integrate their knowledge networks.^20^ Indeed, the ED may be ideally suited for transitioning pre-clerkship learners due to the opportunity to work through undifferentiated patients in a shift-based environment with a consistent mentor.^21^–^23^ Allowing students to practice presenting to an attending faculty member in a clinical but non-graded environment allows students to gain confidence while developing familiarity with the clinical care environment.^5^,^24^,^25^

Of note, students showed substantially increased interest in EM as a potential specialty after completing the CRE. In contrast, a study in 2015 by Lambda et al. showed that a mandatory senior EM clerkship did not significantly change overall students’ perceptions regarding EM.^26^ Based on our findings, early exposure to the ED may be more effective at cultivating interest in EM compared to clerkships later in training.

The CRE was born out of a desire to expose pre-clerkship learners to real patients while developing clinical reasoning skills and establishing the groundwork for forthcoming clinical clerkships. What started as a student-run elective, has served as a framework for the medical school to introduce students to the clinical setting early in their medical education as part of a curricular revision.

## LIMITATIONS

There are several limitations to this study. First, it is unknown if the students who chose not to participate in CRE were different from the CRE participants. Students who are motivated to participate may also be more motivated to study and perform on the CCA. Therefore, it is uncertain whether variables outside participation in the CRE contributed to differences in exam scores.

While both students and physician mentors were provided with the goals and expectations for participation in the program, freedom was given to the faculty to evolve the activities and responsibilities of students as the year progressed. We expect there was variation in the degree of autonomy granted to students, as well as the extent and quality of teaching provided between faculty. Additionally, due to the high volume of students who wished to participate in the CRE, a small number of students were assigned a physician-mentor outside of EM, but their objectives and expectations for the program remained the same as for the rest of the students. An additional limitation may be the self-reported nature of CRE participation. As this course was an ungraded elective, the authors hope that the number of sessions reported by students is accurate; however, this cannot be confirmed.

The M2 CCA and exit survey are an internally developed assessment that has some validity evidence collected, but there are gaps in the collected validity evidence. This is a single institution study, and generalizability of the results is not yet known. Finally, the high ceiling and narrow range of scores may not have allowed us to detect differences between the comparison groups.

## CONCLUSION

Participation in a Clinical Reasoning Elective was associated with increases in students’ scores on a comprehensive clinical assessment at the end of the second year of medical school. Higher examination scores may translate to greater preparedness and decreased stress as these students enter clinical clerkships. In addition, early exposure to the ED, along with longitudinal mentoring by emergency physicians, was associated with greater student interest in EM as a specialty. The framework of the CRE is an effective and popular model to teach history-taking, physical exam, and differential diagnosis skills while transitioning pre-clinical learners to the independent and hands-on learning environment of the clerkships.

## Supplementary Information





## Figures and Tables

**Table:** Impact of student participation in the Clinical Reasoning Elective on M2 CCA domain scores.

	Mean score for all students	Standard deviation	Increase in Score for Every CRE Shift Completed^1^,^2^	Average Increase in Score with Participation in CRE^3^
Abdominal history	91.4%	7.85%	0.295%**	3.24%**
Overall history-taking	90.0%	6.19%	0.212%**	2.68%**
Pulmonary physical exam	94.7%	7.88%	0.218%*	3.47%**
Overall communication	87.8%	5.61%	0.147%*	0.21%
Overall physical exam	95.3%	3.39%	0.088%*	1.36%*

*M2 CCA*, second-year comprehensive clinical assessment; *CRE*, clinical reasoning elective.

1 All other domains were nonsignificant.

2 Unstandardized regression coefficients: p<.05*; p<.01**

3 Dichotomous participation; two-tailed t-test: p<.05; p<.01** This is the difference in average score for dichotomized participation. For example, those that participated had a 3.24% higher average score on abdominal history than those who did not.
