# Supplementary material for: Anything but Shadowing! Early Clinical Reasoning in Emergency Department Improves Clinical Skills
Source: West J Emerg Med. 2017 Dec 22;19(1):177–84. doi: 10.5811/westjem.2017.10.36691 (PMC5785190; doi:10.5811/westjem.2017.10.36691)
Supplement: Supplementary file 1 [file wjem-19-177-s001.docx]

We also tested the hypothesis that our results are driven by demographic variables (i.e., selection) rather than to the effects of CRE.  We re-ran each of the five regression analyses and included demographic variables along with CRE participation as predictors of the M2 CCA domain scores. For these multiple regression analyses, we included age; sex (0=male, 1-female); and four dummy variables representing race/ethnicity (with Whites as the reference group).

∙ Results from multiple regression analysis showed that, even after including demographic variables, CRE participation was statistically significantly associated with higher scores on the Abdominal History component of the exam, *b* = 0.270, *p* < .05. Results also showed that females scored statistically significantly higher than males on the Abdominal History component of the exam, *b* = 3.1, *p* < .05. There were no statistically significant effects of age or race/ethnicity.

∙ Results from multiple regression analysis showed that, even after including demographic variables, CRE participation was statistically significantly associated with higher scores on the History-Taking component of the exam, *b* = 0.183, *p* < .05. Results also showed that females scored statistically significantly higher than males on the History-Taking component of the exam, *b* = 3.0, *p* < .05. There were no statistically significant effects of age or race/ethnicity.

∙ Results from multiple regression analysis showed that, even after including demographic variables, CRE participation was statistically significantly associated with higher scores on the Pulmonary-Physical component of the exam, *b* = 0.214, *p* < .05. There were no statistically significant effects of age, sex, or race/ethnicity.

∙ Results from multiple regression analysis showed that, after including demographic variables, CRE participation was no longer statistically significantly associated with higher scores on the Pulmonary-Physical component of the exam, *b* = 0.104, *p* = 0.15. There were no statistically significant effects of age, sex, or race/ethnicity.

∙ Results from multiple regression analysis showed that, after including demographic variables, CRE participation was no longer statistically significantly associated with higher scores on the Overall Physical component of the exam, *b* = 0.086, *p* = .055. There were no statistically significant effects of age, sex, or race/ethnicity.

∙ In summary, results indicated that demographic variables were not statistically significantly related to participation in the CRE. After statistically controlling for demographic variables, CRE participation continued to be statistically significantly associated with higher scores on the Abdominal History, History-Taking, and Pulmonary-Physical components of the exam. CRE participation was also associated with higher scores on the Overall Communication and Overall Physical components of the exam components of the exam, but these effects were no longer statistically significant when demographic variables were statistically controlled. Thus, although the results do not support the hypothesis that the effects of CRE participation are attributable to demographic variables, findings indicate that the effects of CRE participation are strongest for the Abdominal History, History-Taking, and Pulmonary-Physical components of the exam.
