# Supplementary material for: Anything but Shadowing! Early Clinical Reasoning in Emergency Department Improves Clinical Skills
Source: West J Emerg Med. 2017 Dec 22;19(1):177–84. doi: 10.5811/westjem.2017.10.36691 (PMC5785190; doi:10.5811/westjem.2017.10.36691)
Supplement: Supplementary file 2 [file wjem-19-177-s002.pdf]

Default Question Block

Your name:

[ ]

Your partner's name:

[ ]

Name of faculty that you worked with:

[ ]

Where did you complete your sessions? (select all that apply)

- ☐ Adult Emergency Department
- ☐ Pediatric Emergency Department
- ☐ VA Emergency Department
- ☐ Critical Care / Short Stay / EC3
- ☐ Other Inpatient

How many 4hr sessions did you work with your faculty? (if you attended an entire shift for 8 hrs or more, count as two sessions)

[▾]

On average, how many patients did you see each shift?

[▾]

Clinical reasoning activities:

	How frequently did you and your partner do the following?				How much did this activity contribute to learning?		
	Every session	Most Sessions	Some Sessions	Never	A great deal	A moderate amount	Not at a
Took a patient history without faculty present	○	○	○	○	○	○	○
Performed a physical exam without faculty present	○	○	○	○	○	○	○
Took a patient history while supervised by faculty	○	○	○	○	○	○	○
Performed a physical exam while supervised by faculty	○	○	○	○	○	○	○
Gave an oral presentation of history/physical exam findings	○	○	○	○	○	○	○
Gave a written summary of history/physical exam findings	○	○	○	○	○	○	○
Saw at least one patient relevant to the current sequence we were studying	○	○	○	○	○	○	○
Appreciated abnormal physiology of a condition							

we were currently studying	○	○	○	○	○	○	○
Appreciated abnormal physiology of a condition we had learned in prior sequences	○	○	○	○	○	○	○
Saw at least one patient with a condition we had not covered in coursework	○	○	○	○	○	○	○
Were asked to provide a differential diagnosis for a patient	○	○	○	○	○	○	○
Were asked to provide an assessment and plan for a patient (e.g. which tests should be ordered, possible treatments, etc)	○	○	○	○	○	○	○
Looked at X-Ray, CT, MRI or other radiological tests from a patient that we saw	○	○	○	○	○	○	○
Examined lab tests or blood work from a patient that we saw	○	○	○	○	○	○	○
Other: ______	○	○	○	○	○	○	○
Other: ______	○	○	○	○	○	○	○

Interaction with faculty:

	How frequently did your faculty do the following?				How much did this activity contribute to learning?		
	Every session	Most Sessions	Some Sessions	Never	A great deal	A moderate amount	Not at a
Gave feedback on physical exam technique	○	○	○	○	○	○	○
Gave feedback on oral presentation technique	○	○	○	○	○	○	○
Explained a disease or condition I was unfamiliar with	○	○	○	○	○	○	○
Explained lab values or test results from a patient	○	○	○	○	○	○	○
Explained an X-Ray, CT, MRI or other radiological test from a patient	○	○	○	○	○	○	○
Asked me questions about classic disease presentation / "pimped" me	○	○	○	○	○	○	○
Exposed us to a variety of patients and chief complaints	○	○	○	○	○	○	○
Other: ______	○	○	○	○	○	○	○
Other: ______	○	○	○	○	○	○	○

After an average session with my faculty member, I.... (select all that apply)

- ☐ Felt like I understood a disease process better
- ☐ Recalled a patient that I saw in an ED visit while taking an exam
- ☐ Read / learned more about a condition I saw in the ED after the session was over
- ☐ Followed up / asked about a patient I had seen on a previous session

Overall, the learning experience with my faculty member was:

- ○ Excellent
- ○ Good
- ○ Average
- ○ Poor
- ○ Terrible

As thanks for participating in the Clinical Reasoning Elective, we provide faculty with a letter that they may include in their file for promoti
Please write 2-3 sentences describing how your faculty member helped you learn during this experience.

How could your faculty member have improved your learning experience?

Was it helpful for you to have a partner for this experience?

- Yes
- No
- Unsure

To what extent did the CRE help you:

	A great deal	A lot	A moderate amount	A little	Not at a
Learn sequence material	O	O	O	O	O
Feel comfortable interacting with real patents	O	O	O	O	O
Feel comfortable presenting to faculty	O	O	O	O	O
Feel comfortable asking questions / interacting with attending physicians	O	O	O	O	O
Become familiar with looking at scans / radiological tests	O	O	O	O	O
Understand issues related to insurance coverage and access to care	O	O	O	O	O
Develop differential diagnoses for common chief complaints	O	O	O	O	O

To what extent did the CRE help you prepare for the following components of the M2 CCA?

	A great deal	A lot	A moderate amount	A little	Not at a
History Taking	O	O	O	O	O
Physical Exam	O	O	O	O	O
Communication	O	O	O	O	O
Verbal Presentation	O	O	O	O	O
Case Write-up	O	O	O	O	O
Overall	O	O	O	O	O

Throughout M1 and M2 year we gain clinical reasoning skills through a variety of required and extracurricular activities. Please rank the following activities with 1 being the most valuable and 7 being the least valuable for developing your clinical reasoning skills.

Clinical Reasoning Elective

Clinical Foundations of Medicine (CFM) Small Group Sessions

One-on-one CEP sessions with CFM faculty and standardized patients

Shadowing (set up by individual student)

Shadowing (set up through a student org or interest group)

M1 Standardized Patient Program (SPI)

M1 Sequence Small Groups

M2 Sequence Small Groups

Other

How would you describe your interest in Emergency Medicine as a potential specialty?

	My top choice	Very interested	Somewhat interested	Not at all interes
Before taking the CRE	O	O	O	O
After completing the CRE	O	O	O	O

Overall, the Clinical Reasoning Elective contributed to my learning:

- A great deal
- A lot
- A moderate amount
- A little
- None at all

Please provide any comments/suggestions on how the Clinical Reasoning Elective could be improved:

Powered by Qualtrics
